# Regulators of G-protein signaling, RGS2 and RGS4, inhibit protease-activated receptor 4-mediated signaling by forming a complex with the receptor and Gα in live cells

**DOI:** 10.1186/s12964-020-00552-7

**Published:** 2020-06-09

**Authors:** Yukeyoung Kim, Sungho Ghil

**Affiliations:** Department of Life Science, Kyonggi University, Suwon, 16227 South Korea

**Keywords:** Bioluminescence resonance energy transfer, Cancer progression, Extracellular signal-regulated kinase, Phospholipase

## Abstract

**Background:**

Protease-activated receptor 4 (PAR4) is a seven transmembrane G-protein coupled receptor (GPCR) activated by endogenous proteases, such as thrombin. PAR4 is involved in various pathophysiologies including cancer, inflammation, pain, and thrombosis. Although regulators of G-protein signaling (RGS) are known to modulate GPCR/Gα-mediated pathways, their specific effects on PAR4 are not fully understood at present. We previously reported that RGS proteins attenuate PAR1- and PAR2-mediated signaling through interactions with these receptors in conjunction with distinct Gα subunits.

**Methods:**

We employed a bioluminescence resonance energy transfer technique and confocal microscopy to examine potential interactions among PAR4, RGS, and Gα subunits. The inhibitory effects of RGS proteins on PAR4-mediated downstream signaling and cancer progression were additionally investigated by using several assays including ERK phosphorylation, calcium mobilization, RhoA activity, cancer cell proliferation, and related gene expression.

**Results:**

In live cells, RGS2 interacts with PAR4 in the presence of Gα_q_ while RGS4 binding to PAR4 occurs in the presence of Gα_q_ and Gα_12/13_. Co-expression of PAR4 and Gα_q_ induced a shift in the subcellular localization of RGS2 and RGS4 from the cytoplasm to plasma membrane. Combined PAR4 and Gα_12/13_ expression additionally promoted translocation of RGS4 from the cytoplasm to the membrane. Both RGS2 and RGS4 abolished PAR4-activated ERK phosphorylation, calcium mobilization and RhoA activity, as well as PAR4-mediated colon cancer cell proliferation and related gene expression.

**Conclusions:**

RGS2 and RGS4 forms ternary complex with PAR4 in Gα-dependent manner and inhibits its downstream signaling. Our findings support a novel physiological function of RGS2 and RGS4 as inhibitors of PAR4-mediated signaling through selective PAR4/RGS/Gα coupling.

**Video Abstract**

## Background

G-protein-coupled receptors (GPCR), known as seven-transmembrane receptors based on their structure, are the most abundant class of human cell surface receptors [1]. Ligand binding to GPCRs activates a downstream signaling cascade mediated through heterotrimeric GTP-binding proteins (G-proteins). G-proteins consist of three subunits, specifically, Gα, Gβ, and Gγ. Activation of GPCRs leads to replacement of bound GDP on the Gα subunit with GTP. After activation, bound GTP is hydrolyzed back to GDP via the inherent GTPase activity of the Gα subunit [2]. Gα subunits have been classified into several families (Gα_i/o_, Gα_q/11_, Gα_12/13_, and Gα_s_) [3].

The protease-activated receptor (PAR) is a GPCR with a distinct activation mechanism involving N-terminal cleavage by proteolytic enzymes, such as thrombin, trypsin, and tryptase. The cleaved N-terminal region acts as a tethered ligand that binds to its own receptor [4]. Four PAR families (PAR1–4) have been identified to date [5]. PAR4 couples with two Gα subunits, Gα_q_ and Gα_12/13_, and promotes activation of several effector proteins, including phospholipase C (PLC), mitogen-activated protein kinase, protein kinase C, and Rho small GTPase [6, 7]. Activated PAR4 is mainly involved in platelet aggregation and immune responses [7]. Recent studies have additionally demonstrated overexpression of PAR4 in several malignant cancer types and its involvement in tumor growth and metastasis [8–11].

Regulators of G-protein signaling (RGS) are GTPase-activating proteins (GAP) that inhibit G-protein signaling by inducing GTP hydrolysis of activated Gα (Gα-GTP) [12]. The RGS domain common to these proteins elicits GAP activity by stabilizing Gα in its transition state, thus lowering the required reaction-free energy for GTP hydrolysis and its subsequent return to the Gα-GDP state [13]. Several RGS subfamilies identified based on their amino acid sequences and protein structures are characterized by a shared RGS domain (~ 120 amino acids) that serves as the binding site and confers GAP activity [14]. RGS2 is broadly expressed in both mouse and human tissues and binds Gα_q/11_ to inhibit Gα_q/11_-mediated signaling while RGS4 is enriched in brain and cardiac tissues and interacts with the Gα_i/o_ and Gα_q_ families [15–17].

RGS proteins interact either directly or indirectly with GPCRs to modulate receptor-mediated signaling [15, 18]. Previously, we reported that RGS proteins bind PAR1 and PAR2 together with distinct Gα subunits, and modulate downstream signaling pathways [19–21]. As specified above, PAR4 is involved in various cellular responses and pharmacological effects and its inhibition holds potential therapeutic value in the management of several diseases, including cancer, inflammation, and thrombosis [7, 22]. Although RGS proteins are clearly implicated in inhibition of GPCR activity, the mechanisms underlying suppression of PAR4 are not fully understood at present.

In the current study, we employed the bioluminescence resonance energy transfer (BRET) technique to investigate the network of interactions among PAR4 and RGS proteins in live cells in the presence of Gα proteins, with further focus on the inhibitory effects of RGS proteins on PAR4-mediated signaling. Notably, PAR4 interacted with RGS2 and RGS4, but strictly in a Gα-dependent manner. Additionally, both RGS2 and RGS4 attenuated PAR4-activated downstream signaling and cancer progression. Our collective findings suggest that both RGS proteins selectively modulate PAR4 signaling through Gα-dependent interactions.

## Materials and methods

### Cell culture and transfection

293T and HT29 cell lines (Korean Cell Line Bank, Seoul, Korea) were maintained in Dulbecco’s modified Eagle’s medium (DMEM) supplemented with 100 units∙mL^− 1^ penicillin, 100 mg∙mL^− 1^ streptomycin, 50 mg∙L^− 1^ gentamycin and 10% fetal bovine serum (FBS). All cells were cultured in a 37 °C humidified incubator under 5% CO_2_. Transient transfection of cells was performed using calcium phosphate and Lipofectamine 2000 (Invitrogen, Gaithersburg, MD).

### BRET analysis

PAR4-Venus (Venus-N1-PAR4), a C-terminal Venus-tagged plasmid encoding PAR4, was generated via polymerase chain reaction (PCR)-mediated amplification of pcDNA3.1-PAR4 (cDNA Resource Center, Rolla, MO) using the primer pair 5′-TTA AGC TTT TCA CCA TGT GG-3′(forward) and 5′-AAC GGT ACC AGC CAC TG-3′(reverse). Amplified products were inserted into the Venus-N1 plasmid via the *HindIII* and *KpnI* restriction sites. 293T cells were seeded into six-well cell culture plates (3.5 × 10^5^ cells/well). Cells were transfected with BRET donor (Renilla luciferase-tagged plasmids) and acceptor (Venus-tagged plasmids) along with the indicated plasmids. A constant quantity of total transfected DNA was maintained by adding the appropriate amount of empty plasmid, pcDNA3.1. After 24 h, cells were washed with phosphate-buffered saline (PBS), resuspended in Tyrode’s solution (140 mM NaCl, 5 mM KCl, 1 mM MgCl_2_, 1 mM CaCl_2_, 0.37 mM NaH_2_PO_4_, 24 mM NaHCO_3_, 10 mM HEPES, and 0.1% glucose, pH 7.4) and plated on gray 96-well Optiplates (Perkin Elmer Life Sciences, Waltham, MA). Acceptor expression was determined by measuring fluorescence using a VICTOR-X2 multilabel plate reader (Perkin Elmer Life Sciences, Arlington, IL) with a 485 nm excitation and 530 nm emission filter. For measurement of BRET signals, cells were treated with the luciferase substrate, coelenterazine H (Nanolight Technologies, Pinetop, AZ; final concentration 5 μM), for 2 min. BRET signals were obtained by simultaneous measurement of fluorescence (filter, 530 ± 20 nm) and luciferase signals (filter, 480 ± 20 nm). The BRET ratio was determined by calculating the ratio of light intensity emitted by fluorescence over that emitted by luciferase. The net BRET value was obtained by subtracting the background BRET ratio expressed by the donor alone. The ratio of fluorescence/luminescence units was calculated by dividing the fluorescence value of the BRET acceptor by that of the BRET donor. All measurements were performed in sextuplicate.

### Immunofluorescence and confocal imaging

293T cells attached to poly-D-lysine- and collagen-coated coverslips were transfected with the indicated plasmids. After 48 h, cells were washed once with PBS and subsequently fixed with PBS containing 4% paraformaldehyde at room temperature for 20 min. Cells washed with PBS were blocked for 1 h at room temperature in blocking solution (PBS containing 0.1% Triton X-100, 10% normal goat serum, and 1% bovine serum albumin) and subsequently incubated with blocking solution containing antibodies against HA (1:150 dilution, Biolegend, San Diego, CA) and/or EE (EMD Millipore, Billerica, MA) at room temperature for 1 h. Cells were washed four times for 7 min each and incubated with Alexa Fluor 488- or Alexa Fluor 568-conjugated secondary antibodies (1500; Invitrogen) for 30 min. Following four additional washes for 7 min, cells were mounted with VECTASHIELD (Vector Laboratories, Burlingame, CA) and observed under a Zeiss LSM 700 confocal microscope with 40 × 1.2 numerical aperture objective (Carl Zeiss, Oberkochen, Germany). Pearson correlation coefficient (PCC) was calculated to quantify colocalization between PAR4-Venus and RGS-HA proteins in the presence of Gα. We assigned four square-shaped regions per cell to the cell membrane. PCC was calculated by IMAGE J software (National Institutes of Health, Bethesda, MD) between the stacks of images from two channels.

### Measurement of ERK phosphorylation

293T and HT29 cells seeded into six-well cell culture plates (3.5 × 10^5^ cells/well) were transfected with the indicated plasmids. A constant amount of total transfected DNA was maintained by adding pcDNA3.1. After 24 h, cells were starved in serum-free medium containing DMEM and antibiotics for 48 h, followed by treatment with 20 μM AYPGKF (PAR4-specific agonist peptide, C-terminal amidated; Bachem, Torrance CA) for 7 min. After harvesting of cells with PBTX (PBS containing 1% Triton X-100) containing protease and phosphatase inhibitors, lysates were subjected to immunoblotting with antibodies against p-ERK and total ERK antibodies (Cell Signaling Technology, Danvers, MA).

### Measurement of calcium mobilization

HT29 cells were seeded into 96-well cell culture plates (3.5 × 10^4^ cells/well) and transfected with the indicated plasmids. A constant total amount of total transfected DNA was maintained by adding pcDNA3.1. After 24 h, culture medium was replaced with serum-free medium and cells incubated for an additional 48 h. Cells were treated with 100 μL Fluo-4 dye-loading solution (Fluo-4 assay kit; Abcam, Cambridge, MA) and incubated for 1 h at 37 °C, followed by replacement with Tyrode’s solution. Cells were subsequently treated with 60 μM AYPGKF and calcium mobilization measured for 2000 s at 10 s intervals using a VICTOR-X2 multilabel plate reader with 490 nm excitation and 525 nm emission filters. △F/F_0_ was obtained by subtracting the F_0_ from F and divided by F_0_. F is the intensity of fluorescence emission recorded as the experiment runs. F_0_ is the fluorescence intensity at the start of the experiment.

### Measurement of RhoA activation

BL21 bacterial cells expressing GST-Rhotekin-RBD fusion protein were induced with 0.1 mM IPTG, followed by lysis with PBTX. Lysates were incubated with glutathione-Sepharose 4B beads (GE Healthcare Life Sciences, Munich, Germany) in PBTX for 1 h at 4 °C with gentle rotation, and beads were subsequently washed with PBTX. Extracts of HT29 cell cultured in 100-mm dish expressing the indicated RGS proteins were added to GST-Rhotekin-RBD-bound beads and incubated for 16 h at 37 °C. After washing with PBTX buffer, bound proteins were eluted with SDS sample buffer and subjected to immunoblotting with antibody against RhoA (Santa Cruz Biotechnology, Santa Cruz, CA). The total amount of DNA used for transfection was kept constant with the addition of pcDNA3.1.

### Cell proliferation assay

Cell proliferation was evaluated using the 3-(4,5-dimethylthiazol-2-yl)-2,5-diphenyltetrazolium bromide (MTT) assay. In brief, cells were seeded in 96-well plates at a density of 3.5 × 10^4^ cells/well and transfected with the indicated plasmids. After 48 h, cells were treated with 10 μM AYPGKF for 96 h. The medium was replaced with 200 μL MTT solution (5 mg/mL; Sigma-Aldrich, St Louis, MO), followed by incubation at 37 °C for 4 h. At the end of the incubation period, 100 μL dimethyl sulfoxide was added to solubilize formazan crystals. Absorbance (540 nm) was measured using a SpectraMax Plus 384 microplate reader (Molecular Devices, Sunnyvale, CA). All measurements were performed in triplicate.

### Quantitative reverse transcription PCR (RT-qPCR)

HT29 cells seeded into six-well cell culture plates (3.5 × 10^5^ cells/well) were transfected with the indicated plasmids. A constant total amount of transfected DNA was maintained by adding pcDNA3.1. After 24 h, cells were treated with AYPGKF for 6 h and harvested. Total RNA was extracted using an Easy-Spin Total RNA Extraction kit (Intron Biotechnology, Seongnam, Korea). Reverse transcription was performed with the AccuPower RT PreMix (Bioneer, Daejeon, Korea). Relative mRNA expression was assessed via RT-qPCR on a GENECHECKER PCR System (Genesystem, Daejeon, Korea) using Rapi:chip (Genesystem), Detect Master Mix (Genesystem), and specific gene primer sets (Table 1). Thermocycling conditions were as follows: 95 °C for 30 s (initial denaturation), 50 cycles at 95 °C for 3 s (denaturation), 43–62 °C (specified in Table 1) for 3 s (annealing), and 72 °C for 3 s (extension). Relative mRNA levels were quantified using the Gene Recorder software (Genesystem) after normalization to GAPDH.

**Table 1.** Primer sets

Gene	Forward primer	Reverse primer	Annealing temperature
ATF3	5’-CTG CAG AAA GAG TCG GAG-3’	5’-TGA GCC CGG ACA ATA CAC-3’	53 °C
COX2	5’-GAA TGG GGT GAT GAG CAG TT-3’	5’-CAC AAG GGC AGG ATA CAG C-3’	52 °C
BTF3	5’-AGC TTG GTG CGG ATA TGA-3’	5’-GTG CTT TTC CAT CCA CAG ATT G-3’	55 °C
SNAIL1	5’-GAA AGG CCT TCA ACT GCA AA-3’	5’-TGA CAT CTG AGT GGG TCT GG-3’	56 °C
ZFP91	5’-AGC TAC CAT TTG CCT ACA A-3’	5’-GGG AAA CGG CTG AGA TAG TTT-3’	43 °C
LRH1	5’-GCA TCT TGG GCT GCC TGC AG-3’	5’-CCT TGC CGT GCT GGA CCT GG-3’	62 °C
Sp1	5’-GCC TCC AGA CCA TTA ACC TCA GT-3’	5’-GCT CCA TGA TCA CCT GGG GCA T-3’	61 °C
p21	5’-GTC CGT CAG AAC CCA TGC-3’	5’-GTC GAA GTT CCA TCG CTC A-3’	56 °C
GAPDH	5’-TGG GCT ACA CTG AGC ACC AG-3’	5’-GGG TGT CGC TGT TGA AGT CA-3’	55 °C

### Statistical analysis

Data reflect the standard error of mean of at least three or more independent experiments. Statistical differences were assessed with Student’s *t*-test and expressed using Sigmaplot 10.0 software. Data were considered statistically significant at *P* < 0.05 (* indicates P < 0.05, **P < 0.01, ***P < 0.005, ****P < 0.001). *P*-values determined by comparing data with a second control in the graph are indicated with #.

## Results

### Interactions between PAR4 and either RGS2 or RGS4 in live cells

**Fig. 1:**
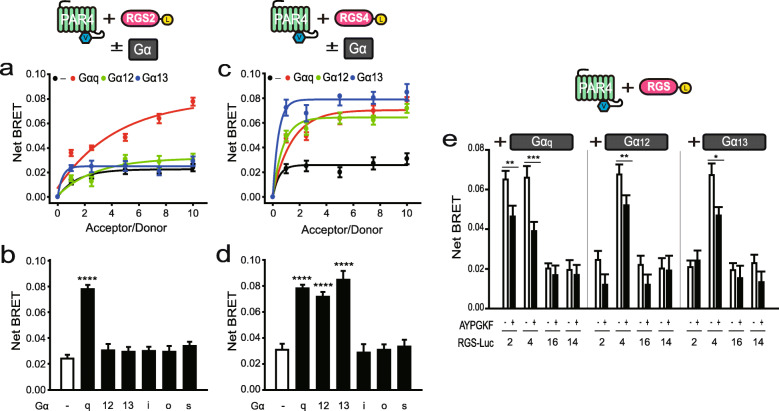
Interactions between PAR4 and either RGS2 or RGS4 in live cells. (Insets of a, c and e) Schematic depiction of fusion and untagged proteins used in the BRET experiment. **a**, **c** BRET analysis of 293 T cells co-transfected with PAR4-Venus (0, 0.1, 0.25, 0.5, 0.75, 1.0 μg), either RGS2-Luc (0.1 μg) (**a**) or RGS4-Luc (0.1 μg) (**c**), and 0.5 μg of the indicated untagged Gα^EE^. **b**, **d** BRET analysis of 293 T cells transfected with PAR4-Venus (1.0 μg), either RGS2-Luc (0.1 μg) (**b**) or RGS4-Luc (0.1 μg) (**d**), and 0.5 μg of the indicated untagged Gα^EE^. **e** BRET analysis of cells were transfected with 1 μg PAR4-Venus, 0.1 μg RGS-Luc, and 0.5 μg indicated untagged Gα^EE^ in the presence and absence of AYPGKF, a PAR4 agonist. *P < 0.05, **P < 0.01, ***P < 0.001, ****P < 0.0001, compared to controls. All BRET results are representative of at least three independent experiments

To determine whether PAR4 interacts with RGS2 in live cells, we performed BRET analysis using PAR4-Venus (PAR4-Ven) and RGS2-Luciferase (RGS2-Luc) expression plasmids. For this experiment, 293T cells were transfected with PAR4-Ven (0–1.0 μg) and RGS2-Luc (0.1 μg) as the acceptor and donor, respectively (Fig. 1a). Analysis of BRET signals revealed that PAR4 did not interact with RGS2 alone (black line). In view of the finding that PAR4 couples with Gα_q_ and Gα_12/13_ families, we further investigated the effects of Gα_q_ and Gα_12/13_ on these interactions [6, 23]. Notably, PAR4-RGS2 interactions were enhanced upon expression of Gα_q_ (red line) but not Gα_12/13_ (green and blue lines, respectively). Examination of the effects of other Gα subunits on PAR4-RGS2 interactions disclosed no alterations in BRET signals in the presence of Gα_i_, Gα_o_ or Gα_s_ (Supplementary Figure S1). Each end-point values (experimental conditions: 1.0 μg PAR4-Ven, 0.1 μg RGS2-Luc, and 0.5 μg Gα) of the net BRET signal is presented in Fig. 1b.

To investigate whether PAR4 also interacts with RGS4 and whether Gα subunits contribute to this interaction, BRET analysis was performed using 293T cells transfected with PAR4-Ven (0–1.5 μg), RGS-Luciferase (RGS4-Luc, 0.1 μg), and an indicated Gα subunit (0.5 μg) (Fig. 1c). PAR4 did not interact with RGS4 alone but co-expression with Gα_q_ or Gα_12/13_ promoted interactions between PAR4 and RGS4. We additionally investigated the potential contribution of other Gα subunits to PAR4-RGS4 interactions. No strong BRET signals were evident between PAR4 and RGS4 in the presence of Gα_i_, Gα_o_ or Gα_s_ (Supplementary Figure S2). Each end-point values of the net BRET signal is presented in Fig. 1d.

The effects of the PAR4 agonist, AYPGKF, on PAR4-RGS-Gα interactions, were examined. BRET analysis was conducted using 293T cells transfected with PAR4-Ven (1.0 μg), the indicated RGS-Luc (0.1 μg), and Gα (0.5 μg), in the presence and absence of the agonist (Fig. 1e). In the presence of Gα_q_, PAR4 displayed interactions with both RGS2 and RGS4 and specifically with RGS4 in the presence of Gα_12/13_. Notably, binding was significantly inhibited by PAR4 activation. No BRET signals were observed in cells co-expressing PAR4-Ven and either RGS16-Luc or RGS14-Luc belonging to the B/R4 and non-B/R4 RGS subfamilies, respectively, in the presence of Gα_q_ and Gα_12/13_, regardless of PAR4 agonist stimulation. We further determined whether other Gα proteins contribute to PAR4-RGS16 or PAR4-RGS14 interactions. BRET analysis of 293T cells transfected with 1.0 μg PAR4-Ven, 0.1 μg RGS-Luc and 0.5 μg Gα (Supplementary Figure S3) disclosed no interactions of PAR4 with RGS16 or RGS14, regardless of the presence of Gα. Our collective findings suggest that PAR4 interacts with both RGS2 and RGS4 in the presence of Gα_q_ and specifically with RGS4 in the presence of Gα_12/13_. Moreover, these interactions are dissociated upon PAR4 activation.

### Membrane localization of RGS proteins in the presence of PAR4 and Gα

**Fig. 2:**
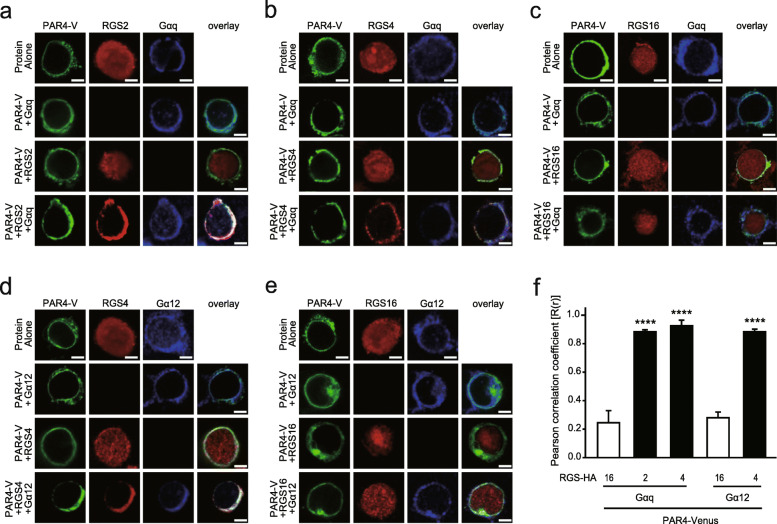
Subcellular localization of RGS proteins in the presence of PAR4 and Gα proteins. **a**-**e** 293 T cells were transfected with the indicated combinations of PAR4-Venus (PAR4-V, 1.0 μg, a-e), RGS2-HA (1.0 μg, **a**), RGS4-HA (1.0 μg, **b** and **d**), RGS16-HA (1.0 μg, **c** and **e**), Gα_q_^EE^ (1.0 μg, **a**–**c**), and Gα_12_^EE^ (1.0 μg, **d**–**e**). After immunofluorescence analysis with antibodies against HA and EE, cells were observed under a confocal microscope. Scale bar: 10 μm. **f** Pearson correlation coefficient analysis was used for quantifying colocalization between PAR4-Venus and RGS-HA proteins in the presence of Gα. ****P < 0.0001, compared to each control. Results are representative of at least three independent experiments

To ascertain whether RGS2 and RGS4 colocalize with PAR4 and Gα_q_, 293T cells were co-transfected with PAR4-Ven and Gα_q_^EE^ (EE epitope-tagged Gα_q_) together with either RGS2-HA, RGS4-HA or RGS16-HA, and analyzed via immunohistochemistry using antibodies against HA and EE (Fig. 2a-c). Confocal microscopy images revealed that RGS2, RGS4, and RGS16 mainly localized to the cytoplasm, even in the presence of PAR4. Expression of Gα_q_ induced translocation of RGS2 and RGS4, but not RGS16, to the plasma membrane.

Next, we investigated whether RGS4 colocalizes with PAR4 and Gα_12_ by co-transfecting cells with PAR4-Ven and Gα_12_^EE^ (EE epitope-tagged Gα_12_) together with either RGS4-HA or RGS16-HA (Fig. 2d and e). Results of immunohistochemical staining showed that co-expression of PAR4 and Gα_12_ led to significant redistribution of RGS4 from the cytoplasm to plasma membrane. However, RGS16 remained in the cytoplasm, even in the presence of both PAR4 and Gα_12_.

We further quantified the extent of colocalization of PAR4-Ven and RGS-HA proteins in the presence of Gα with the aid of PCC analysis (Fig. 2f). PCC values closer to zero corresponded to lower correlations between the two proteins. In the presence of Gα_q_, PAR4 colocalized with RGS2 and RGS4, but not RGS16. Additionally, Gα_12_ induced a significant increase in colocalization between PAR4 and RGS4, but not RGS16.

### Effects of RGS proteins on PAR4-mediated signaling

**Fig. 3:**
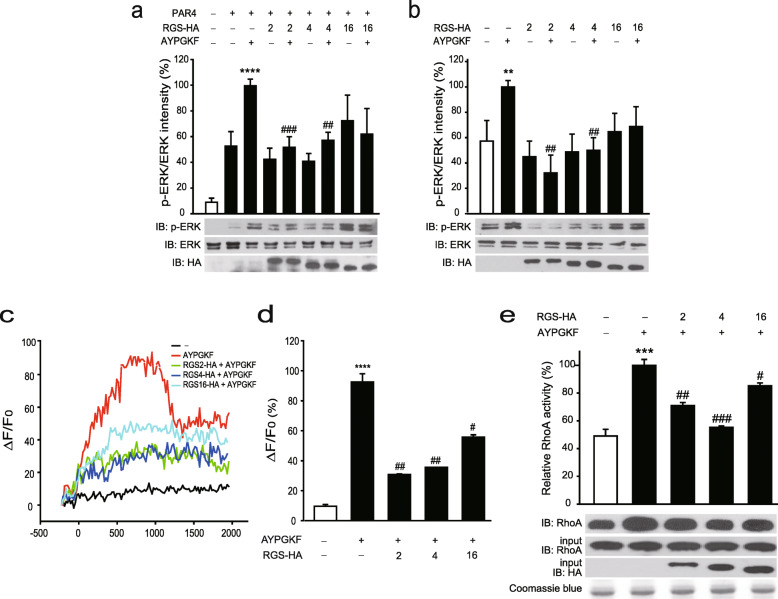
Inhibitory effects of RGS2 and RGS4 on PAR4/Gα_q_- and PAR4/Gα_12/13_-mediated signaling. **a** 293 T cells were transfected with PAR4 (1.0 μg) together with RGS2-HA (1.0 μg), RGS4-HA (1.0 μg) or RGS16-HA (1 μg), as indicated. **b** HT29 cells were transfected with RGS2-HA (1.0 μg), RGS4-HA (1.0 μg) or RGS16-HA (1.0 μg). **a**–**b** After transfection, cells were stimulated with 20 μM AYPGKF for 7 min and immunoblotting performed on cell lysates using antibodies against p-ERK and total ERK. Expression of RGS proteins was confirmed using antibodies against HA. Densitometry analysis was conducted to determine the relative band intensity of p-ERK/ERK. **c**–**d** HT29 cells were transfected with RGS2-HA (0.1 μg), RGS4-HA (0.1 μg) or RGS16-HA (0.1 μg) and treated with Fluo-4 dye-loading solution for 1 h. Fluo-4 solution was replaced with Tyrode’s solution containing 60 μM AYPGKF and intracellular calcium levels measured for 2000 s at 10 s intervals (**c**). Relative intracellular calcium levels measured at 960 s are shown in (**d**). **e** Beads charged with bacterially expressed GST-Rhotekin-RBD were incubated with extracts of HT29 cells expressing 10 μg plasmid encoding RGS2-HA, RGS4-HA or RGS-HA16 in the presence and absence of 20 μM AYPGKF. Bound proteins were immunoblotted with anti-RhoA antibodies. HT29 cell extracts (10%) were used as the loading input for the GST pulldown assay and immunoblotted with anti-RhoA and -HA antibodies. Coomassie blue staining was used to estimate the levels of GST-Rhotekin-RBD fusion proteins. *P < 0.05, **P < 0.01, ***P < 0.001, ****P < 0.0001, compared to unstimulated control, and #*P* < 0.05, ##*P* < 0.01, ###*P* < 0.001, ####*P* < 0.0001, compared to AYPGKF-stimulated control. The results are representative of at least three independent experiments

Next, we investigated the effects of RGS proteins on PAR4-activated ERK phosphorylation [24]. Briefly, 293T cells were co-transfected with plasmids encoding PAR4 and RGS-HA proteins and treated with 20 μM of AYPGKF, which was the most effective concentration in our system (Supplementary Figure S4a), as indicated (Fig. 3a). Cell extracts were subjected to immunoblotting with antibodies against p-ERK and ERK. Band intensity analyses revealed an increase in ERK phosphorylation under conditions of PAR4 expression, which was markedly upregulated in the presence of AYPGKF. ERK phosphorylation induced by activated PAR4 was significantly downregulated by RGS2 and RGS4. RGS16 expression also inhibited PAR4 activation, but not to a significant extent (*P* = 0.10). The effects of RGS proteins on PAR4-mediated ERK phosphorylation were further examined in HT29 colon cancer cells, which normally express PAR4 [10]. For this experiment, HT29 cells transfected with RGS-HA proteins and treated with 20 μM of AYPGKF, which was the most effective concentration in our system (Supplementary Figure S4b) were subjected to immunoblot analysis (Fig. 3b). Treatment with AYPGKF promoted ERK phosphorylation, which was significantly reduced upon RGS2 and RGS4 expression. RGS16 expression also induced a decrease in PAR4 activation, but not to a significant extent (*P* = 0.13).

PAR4 is reported to couple with Gα_q_ and Gα_12/13_ to modulate various downstream effectors, including PLCβ and RhoA. In view of this finding, we determined whether RGS proteins affect PAR4/Gα_q_-mediated signaling. Calcium mobilization responses were assessed by evaluating the relative fluorescence of Fluo-4 in the presence of 60 μM of AYPGKF, which was the most effective concentration in our system (Supplementary Figure S4c) and RGS-HA proteins in HT29 cells (Fig. 3c and d). Stimulation with AYPGKF resulted in increased intracellular calcium levels. However, cells expressing RGS-HA proteins contained lower calcium levels despite AYPGKF stimulation (Fig. 3c). Intracellular calcium levels measured after 960 s of AYPGKF stimulation are presented in Fig. 3d. Notably, the increase in calcium levels induced by AYPGKF were significantly abolished upon RGS2, RGS4, and RGS16 expression.

To determine the effects of RGS proteins on PAR4-mediated Gα_q_ and Gα_12/13_ signaling, RhoA activity, known to be influenced by both Gα_q_- and Gα_12/13_ [25, 26], was measured using a GST pulldown assay. Bacterially purified GST-Rhotekin-RBD fusion proteins were incubated with HT29 cell extracts expressing RGS-HA proteins in the absence or presence of 20 μM of AYPGKF, which was most effective concentration in our system (Supplementary Figure S4d) (Fig. 3e). AYPGKF induced an increase in RhoA activity, which was significantly attenuated by RGS2, RGS4, and RGS16. Specifically, RGS2 and RGS4 inhibited the activities of PAR4-mediated Gα_q_ and Gα_12/13_ downstream molecules, including ERK, PLCβ, and RhoA, while RGS16 induced downregulation of PLCβ and RhoA activities.

### Effects of RGS proteins on PAR4-mediated cancer cell proliferation and gene expression

**Fig. 4:**
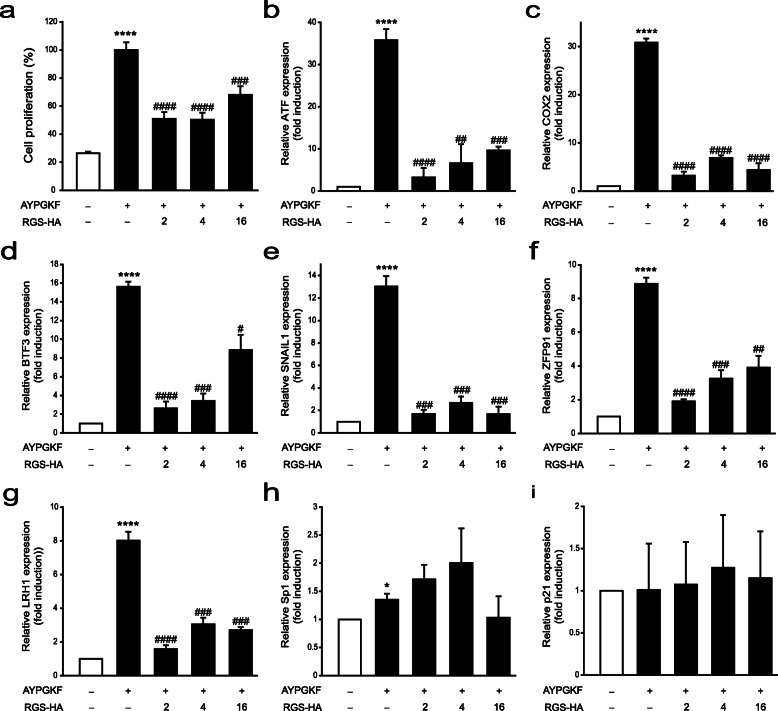
Effects of RGS proteins on PAR4-mediated cancer progression. **a** HT29 cells were transfected with RGS2-HA (0.1 μg), RGS4-HA (0.1 μg) or RGS16-HA (0.1 μg), as indicated, and treated with 10 μM AYPGKF for 96 h. Cell proliferation was evaluated using the MTT assay. **b**-**i** HT29 cells transfected with RGS2-HA (1 μg), RGS4-HA (1 μg), or RGS16-HA (1 μg), as indicated, were treated with 10 μM AYPGKF for 6 h. The relative expression levels of cancer progression-related genes, including ATF3 (**b**), COX2 (**c**), BTF3 (**d**), SNAIL1 (**e**), ZFP91 (**f**), LRH1 (**g**), Sp1 (**h**) and p21 (**i**), were measured using RT-qPCR with specific primer sets (Table 1). Relative expression levels were normalized to that of GAPDH. *P < 0.05, **P < 0.01, ***P < 0.001, ****P < 0.0001, compared to unstimulated control, and #*P* < 0.05, ##*P* < 0.01, ###*P* < 0.001, ####*P* < 0.0001, compared to AYPGKF-stimulated control. The results are representative of at least three independent experiments

Since PAR4 activation induces progression of various cancer types, including colon cancer [8, 9], we examined whether PAR4-induced proliferation is inhibited by RGS in HT29 cells. Significant elevation of cell proliferation was observed upon treatment with 10 μM AYPGKF, which was the most effective concentration in our system (Supplementary Figure S4e) for 96 h, which was markedly reduced in the presence of RGS proteins, including RGS2, RGS4, and RGS16 (Fig. 4a).

Experiments were performed to ascertain the effects of PAR4 and RGS proteins on several genes related to colon cancer progression, including activating transcription factor 3 (ATF3), cyclooxygenase 2 (COX2), basic transcription factor 3 (BTF3), SNAIL1, zinc finger protein 91 (ZFP91), liver receptor homolog 1 (LRH1), specificity protein 1 (Sp1), and p21. All the above genes are known to promote colon cancer progression, with the exception of p21, which acts as a tumor suppressor. HT29 cells were treated with 10 μM AYPGKF for 6 h and gene expression analyzed via RT-qPCR (Fig. 4b–i). Expression of all genes, except p21, was significantly augmented under conditions of PAR4 activation. Increased gene expression was abolished upon RGS2, RGS4, and RGS16 expression. However, Sp1 expression was not altered by expression of RGS proteins. Our results suggest that PAR4 activation contributes to cancer progression, which is attenuated in the presence of RGS2, RGS4, and RGS16.

## Discussion

In this study, we investigated interactions of RGS2 and RGS4 with PAR4 in live cells and determined their effects on PAR4/Gα-mediated signaling. Our data showed that RGS2 binds PAR4 in the presence of Gα_q_ while RGS4 binding to PAR4 requires either Gα_q_ or Gα_12/13_. RGS16 failed to interact with PAR4, regardless of the presence or absence of Gα subunits. In addition, co-expression of PAR4 and Gα_q_ induced a shift in the subcellular localization of RGS2 and RGS4 from the cytoplasm to plasma membrane. Combined PAR4 and Gα_12/13_ expression additionally promoted translocation of RGS4 from the cytoplasm to the membrane. In contrast, subcellular localization of RGS16 was not altered upon co-expression of PAR4 with either Gα_q_ or Gα_12/13_. Our collective results support the formation of specific ternary complexes (PAR4-RGS2-Gα_q_, PAR4-RGS4-Gα_q_ and PAR4-RGS4-Gα_12/13_) in live cells. Furthermore, RGS2 and RGS4 inhibited PAR4/Gα-mediated signaling, as determined from analysis of PAR4-activated ERK phosphorylation, calcium mobilization, and RhoA activity. RGS16 inhibited PAR4-activated calcium mobilization and RhoA activity to an extent but failed to affect ERK phosphorylation. Finally, PAR4-mediated HT29 cancer cell progression was significantly inhibited by RGS2, RGS4 and RGS16, as determined based on PAR4-activated cell proliferation and expression patterns of cancer progression-related genes. In view of these findings, we propose that RGS2 and RGS4 inhibit PAR4-mediated signaling by forming distinct Gα_q_ and Gα_12/13_-dependent complexes in live cells.

Although it has been known that Gα_q_ and Gα_12/13_ endogenously expressed in 293T cells [27], very weak BRET signal was shown in PAR4-Ven and RGS-Luc in the absence of untagged Gα (Fig. 1). We believe that the concentration of endogenous Gα was not enough to elevate the BRET signal.

RGS proteins can interact with GPCRs either directly or indirectly to modulate their function. For instance, the localization, stability, and activity of RGS7 are enhanced by interaction with the orphan receptor, GPR158/179, and Gβ_5_ subunit [28, 29]. RGS14 interacts with the α_2_-adrenergic receptor (α_2_-AR) in the presence of Gα_i_, which is dissociated by receptor activation [30]. RGS20 forms a complex with a melatonin receptor (MT) and Gα_i_, which modulates MT-mediated K^+^ channel activity. According to a model proposed by Maurice et al. [31], one RGS20 and one Gα_i_ subunit bind separately to an MT dimer to form a tetramer. RGS2 interacts with an intracellular loop of AR and modulates Gα-mediated activation of downstream molecules, including adenylyl cyclase and PLC [18, 32]. Earlier, our group showed that RGS proteins inhibit PAR1- and PAR2-mediated downstream signaling through formation of ternary complexes with the receptors and distinct Gα proteins [19–21]. Consistently, data from the current study showed that RGS2 and RGS4 form complexes with PAR4 and distinct Gα subunits, resulting in inhibition of PAR4-mediated downstream signaling. Therefore, we hypothesize that GPCRs function as a molecular hub possessing Gα and RGS proteins in the plasma membrane that regulate G-protein signaling. In support of this theory, our confocal imaging analyses showed that RGS2 and RGS4 translocate from the cytoplasm to the membrane in the presence of PAR4 and Gα whereas RGS16 remains in the cytoplasm under the same conditions.

In our experiments, RGS2 inhibited PAR4-induced downstream signaling events, including ERK phosphorylation, calcium mobilization, RhoA activity, and cancer progression-related gene expression, suggesting that RGS2 forms a ternary complex with PAR4 and Gα_q_, PAR4-RGS2-Gα_q_, that acts to inhibit PAR4/Gα_q_ signaling. RGS2 further suppressed PAR4-mediated RhoA activation, as shown in Fig. 3e. RGS2 is reported to specifically inhibit Gα_q_. Notably, RhoGEF has also been identified as one of the downstream molecules of Gα_q_ [25, 26]. Accordingly, we propose that inhibition of RhoA activity by RGS2 is attributable to RhoGEF suppression via Gα_q_. Our results additionally indicate that RGS4 inhibits PAR4-activated downstream signaling through formation of a PAR4-RGS4-Gα_q_ complex. Although our BRET and confocal microscopy results support the potential formation of a PAR4-RGS4-Gα_12/13_ complex, RGS4 mainly binds and inhibits Gα_q_, giving rise to the theory that the inhibitory effects of RGS4 on PAR4-activated downstream events are predominantly mediated through Gα_q_. Additionally, RGS16 blocked PAR4-mediated calcium mobilization and RhoA activity. These effects of RGS16 were exerted via Gα_q_ and Gα_12/13_ regardless of their interactions with PAR4, but were generally less pronounced than those of RGS2 and RGS4, as shown in Figs. 3 and 4. We speculate that the inhibitory effects of RGS proteins on PAR4 are enhanced through formation of PAR4-RGS-Gα ternary complexes. However, the possibility that the specificity of RGS16 for PAR4-mediated signaling is inherently lower than that of RGS2 and RGS4 cannot be discounted.

Although RGS16 failed to PAR4 interaction and membrane translocation, it inhibited PAR4-mediated calcium and RhoA signaling. It has been known that RGS16 directly interacts with Gα_i_ and Gα_q_, and inhibits their signaling [33]. Therefore, we believe that RGS16 would directly interact with Gα without PAR4 and prohibit Gα signaling. Although, in our confocal images (Fig. 2c and e), most RGS16 localized in cytoplasm in the presence of PAR4 and Gα, small amounts of RGS16 appeared on cell membrane which might interact with Gα proteins. These findings are similar to those of our previous studies. RGS2 forms a ternary complex with PAR1 and Gα_q_, and inhibits PAR1/Gα_q_ signaling, whereas it blocks PAR1/Gα_i/o_ signaling without PAR1 interaction [21]. RGS8 also inhibits PAR1/Gα_o_ signaling with forming trimeric complex, whereas PAR1/Gα_i_ signaling is downregulated by RGS8 without PAR1 interaction. Notably, RGS8 is observed in both cell membrane and cytoplasm in the presence of PAR1, RGS8 and Gα_i_ [20]. Furthermore trimeric PAR2-RGS16-Gα_i_ complex inhibits PAR2/Gα_i_ signaling, whereas RGS16 suppresses Gα_o_ signaling without PAR2 interaction. Also in this case, RGS8 is found in both cytoplasm and membrane in the presence of PAR2, RGS16 and Gα_o_ [19].

The net BRET values of PAR4-Ven-RGS2-Luc-Gα_q_, PAR4-Ven-RGS4-Luc-Gα_q_ and PAR4-Ven-RGS4-Luc-Gα_12/13_ were attenuated by over ~ 30% in the presence of AYPGKF, indicative of dissociation of the ternary complex. Several lines of evidence support the theory that ternary complexes formed by GPCRs, Gαs and G-protein regulators are dissociated by specific agonists. Activators of G-protein signaling (AGS) are biological regulators that influence signal transfer from receptor to G-proteins through guanine nucleotide binding and hydrolysis. Dissociation of specific protein complexes formed by GPCRs, Gαs and AGS under conditions of agonist stimulation has been reported [34, 35]. The α_2_-AR forms a ternary complex with Gα_i_ and RGS14 in its resting state. The Gα_i_-RGS14 complex is dissociated from the receptor in the presence of a specific agonist and preserved after receptor activation [30]. The finding that PAR4-activated Gα_q_ signaling is blocked by RGS proteins after receptor activation supports the possibility that the RGS-Gα_q_ dimer is also preserved but this requires further validation. Previously, we reported that PAR1-RGS2-Gα_q/11_ complex formation is reinforced by receptor activation [21] whereas PAR1-RGS8-Gα_o_ and PAR2-RGS16-Gα_o_ complexes are not affected [19, 20]. Therefore, association or dissociation of the ternary complex in relation to receptor activation appears dependent on the activities of individual components (GPCR, RGS, and Gα).

PAR4 is implicated in various cellular pathophysiologies, including inflammation, thrombosis, pain, and cancer. However, the potential association of PAR4 with cancer progression is controversial. Upregulation of PAR4 is reported to induce apoptosis in prostate and esophageal cancer cells [36–38] and suppression of expression shown to trigger aggressive gastric cancer [39] and poor prognosis and recurrence of breast cancer [40, 41]. However, PAR4 activation mainly induces progression of colon cancer. Agonist stimulation of PAR4 promotes cell proliferation through an increase in ERK phosphorylation and activation of epidermal growth factor receptor B-2 in the HT29 colon cancer cell line [10]. Expression levels of PAR4 mRNA and protein in colon cancer tissues are significantly higher than those in normal tissues. PAR4 activation is reported to induce cell proliferation, invasion, and migration of HT29 cells, which are markedly downregulated upon its knockdown [8, 9]. As expected, PAR4 activation led to a significant increase in cell proliferation in our experiments, which was markedly inhibited by RGS2, RGS4, and RGS16.

Molecular mechanisms underlying the inhibitory effects of RGS proteins on PAR4-mediated cell proliferation can be determined by assessment of gene expression levels during colon cancer progression. Stress-inducible transcription factor, ATF3, responds to a variety of stress signals, including toxins, cytokines, and growth factors. ATF3 plays a dichotomous role in determining cell fate depending on the cell type. The molecule not only induces cell proliferation and oncogenesis [42, 43] but also promotes apoptotic cell death through interactions with p53 [44, 45]. In HT29 cells, ATF3 has been shown to promote tumor growth, invasion, and migration [46, 47]. COX2 stimulates the prostaglandin E_2_ pathway, in turn, inducing tumor growth and invasion, and inhibition of apoptosis [48]. In a colon cancer cell line, chemoresistant tumors with high COX2 expression levels showed aggressive growth rates. Moreover, COX2 inhibition attenuated the proliferative and invasive activities of these tumors [49, 50]. In the current study, ATF3 and COX2 mRNA expression were increased by over ~ 30-fold upon PAR4 activation, compared to the control group, which was abolished by RGS2, RGS4, and RGS16 expression.

BTF3 was initially identified as a member of the general transcription machinery that forms a stable complex with RNA polymerase [51]. The protein is overexpressed in various cancer cell types, including glioma, hepatocarcinoma, pancreatic ductal adenocarcinoma, and colon cancer cells [52]. Recent reports have shown that downregulation of BTF3 attenuates tumorigenesis in colon cancer cells [53, 54]. SNAIL1 is a key transcription factor in the early epithelial-to-mesenchymal transition period, the initial and critical timeframe for metastasis [55]. Enhanced SNAIL1 expression is associated with more aggressive phenotype, poorer clinical outcomes, and more frequent distant metastases in colon cancer [56]. ZFP91, a nuclear protein containing zinc-finger domains, functions as a transcription factor [57]. Upregulation of ZFP91 is reported to enhance tumorigenesis in a colon cancer cell line through promotion of hypoxia-inducible factor-1 gene expression [58]. LRH1 (also known as NR5A2), a member of the nuclear receptor family, was initially identified in mouse liver [59]. Since then, involvement of LRH1 in the development of various malignant tumors, including breast, liver, gastric, colon and pancreatic cancer, has been documented. LRH1 is highly expressed in tissue samples of colon cancer patients, compared to normal tissue, and correlated with the overall survival rate [60]. Knockdown of LRH1 has been shown to attenuate cell proliferation and induce changes in cell cycle patterns and gene expression profiles [61]. Here, PAR4 activation promoted BTF3, SNAIL, ZFP91, and LRH1 mRNA levels by 8–15-fold relative to the control, and this observed increase was inhibited by RGS proteins.

Sp1, a transcription factor involved in early development, is implicated in colon cancer cell growth and progression [62]. Interestingly, PAR4 activation induced only a slight increase in Sp1 mRNA expression in our experiments, indicating no strong association between the molecules. p21 is a representative tumor suppressor in colon cancer [63]. As expected, PAR4 activation did not affect p21 mRNA expression in this study.

## Conclusion

The current investigation focused on the binding properties of PAR4, Gα, and RGS proteins, including RGS2 and RGS4, in live cells. We additionally determined the effects of RGS proteins on PAR4-mediated signaling. RGS2 and RGS4 proteins formed specific ternary complexes with PAR4 and Gα_q_ that acted to inhibit PAR4/Gα_q_ signaling. The RGS16 protein inhibited PAR4/Gα_q_ signaling in the absence of interactions with PAR4. Furthermore, PAR4 activation promoted cell proliferation and cancer-related gene expression, which were attenuated by RGS2, RGS4, and RGS16. Our findings suggest that PAR4 functions as a molecular hub with specific RGS and Gα proteins to modulate downstream signaling. To our knowledge, this is the first study to demonstrate that RGS2, RGS4, and RGS16 proteins inhibit PAR4/Gα_q_-mediated signaling and cancer progression.

## Supplementary information

**Additional file 1: Figure S1.** Interactions between PAR4 and RGS2 in the presence of Gα_i/o_ (a) and Gα_s_ (b) in live cells. (inset) Schematic depiction of fusion and untagged proteins used for the BRET experiment. 293 T cells co-transfected with RGS2-Luc (0.1 μg) and PAR4-Venus (0, 0.1, 0.25, 0.5, 0.75, 1 μg) together with 0.5 μg of the indicated untagged Gα^EE^ were subjected to BRET analysis. All results are representative of at least three independent experiments.

**Additional file 2: Figure S2.** Interactions between PAR4 and RGS4 in the presence of Gα_i/o_ (a) and Gα_s_ (b) in live cells. (Inset) Schematic depiction of fusion and untagged proteins used for BRET. 293T cells co-transfected with RGS4-Luc (0.1 μg) and PAR4-Venus (0, 0.1, 0.25, 0.5, 0.75, 1 μg) together with 0.5 μg indicated untagged Gα^EE^ were subjected to BRET analysis. All results are representative of at least three independent experiments.

**Additional file 3: Figure S3**. Interactions between PAR4 and either RGS16 (a) or RGS14 (b) in the presence of Gα in live cells. (Inset) Schematic depiction of fusion and untagged proteins used for BRET. 293T cells co-transfected with PAR4-Venus (1 μg) and either RGS16-Luc (0.1 μg) or RGS14-Luc (0.1 μg) together with 0.5 μg indicated untagged Gα^EE^ were subjected to BRET analysis. All results are representative of at least three independent experiments.

**Additional file 4: Figure S4.** Establishment of effective PAR4 agonist concentration (a) 293 T cells were transfected with PAR4 (1.0 μg). After transfection, cells were stimulated with 0, 7, 10, 20, 30 μM of AYPGKF for 7 min and immunoblotting was performed on cell lysates using antibodies against p-ERK and total ERK. (b) HT29 cells were stimulated with 0, 7, 10, 20, 30 μM of AYPGKF for 7 min and immunoblotting was performed on cell lysates using antibodies against p-ERK and total ERK. (c) HT29 cells were treated with Fluo-4 dye-loading solution for 1 h. Fluo-4 solution was replaced with Tyrode’s solution containing 0, 10, 30, 60, 90, 120, 150, 180 μM of AYPGKF and intracellular calcium levels measured for 2000 s at 10s intervals. (d) Beads charged with bacterially expressed GST-Rhotekin-RBD were incubated with extracts of HT29 cells which were stimulated with 0, 7, 10, 20, 30 μM of AYPGKF for 7 min. Bound proteins were immunoblotted with anti-RhoA antibodies. HT29 cell extracts (10%) were used as the loading input for the GST pulldown assay and immunoblotted with anti-RhoA antibodies. (e) HT29 cells were treated with 0, 7, 10, 20, 30 μM of AYPGKF for 96 h. Cell proliferation was evaluated using the MTT assay.

## Data Availability

The data set supporting the results of this article is included within the article and its additional files.

## Abbreviations

ATF3: Activating transcription factor 3
BRET: Bioluminescence resonance energy transfer
BTF3: Basic transcription factor 3
COX2: Cyclooxygenase 2
DMEM: Dulbecco’s modified Eagle’s medium
FBS: Fetal bovine serum
GAP: GTPase-activating protein
GPCRs: G-protein-coupled receptors
G-protein: Heterotrimeric GTP-binding protein
LRH1: Liver receptor homolog 1
PAR: Protease-activated receptor
PBS: Phosphate-buffered saline
PBTX: PBS containing 1% Triton X-100
PCC: Pearson correlation coefficient
PCR: Polymerase chain reaction
PLC: Phospholipase C
RGS: Regulators of G-protein signaling
RT-qPCR: Quantitative reverse transcription PCR
Sp1: Specificity protein 1
ZFP91: Zinc finger protein 91
